# A youth empowerment intervention to prevent childhood obesity: design and methods for a cluster randomized trial of the H_2_GO! program

**DOI:** 10.1186/s12889-021-11660-5

**Published:** 2021-09-15

**Authors:** Monica L. Wang, Linda S. Sprague Martinez, Janice Weinberg, Selenne Alatorre, Stephenie C. Lemon, Milagros C. Rosal

**Affiliations:** 1 Department of Community Health Sciences, Boston University School of Public Health, 801 Massachusetts Avenue, Boston, MA 02118 USA; 2 Office of Narrative, Boston University Center for Antiracist Research, Boston, MA 02215 USA; 3 Department of Health Policy and Management, Harvard T.H. Chan School of Public Health, Boston, MA 02215 USA; 4 Macro Department, Boston University School of Social Work, 264 Bay State Rd, Boston, MA 02215 USA; 5 Center for Social Work Innovation in Health, Boston University School of Social Work, 801 Massachusetts Avenue, Boston, MA 02118 USA; 6 Department of Biostatistics, Boston University School of Public Health, 801 Massachusetts Avenue, Boston, MA 02118 USA; 7 Division of Preventive and Behavioral Medicine, Department of Population and Quantitative Health Sciences, University of Massachusetts Medical School, 368 Plantation St, Worcester, MA 01655 USA

**Keywords:** Childhood obesity, Youth empowerment, Cluster randomized trial, Sugar-sweetened beverage consumption, Design and methods

## Abstract

**Background:**

Reducing sugar-sweetened beverage (SSB) consumption is a promising dietary target for childhood obesity prevention. This paper describes the design and methods of a cluster randomized trial of H_2_GO!, a youth empowerment intervention to prevent childhood obesity through reducing SSB consumption among a low-income, ethnically diverse sample of youth.

**Methods:**

This cluster randomized controlled trial is an academic-community partnership with the Massachusetts Alliance of Boys and Girls Clubs (BGC). Ten BGC sites will be randomly assigned to the H_2_GO! intervention or a wait-list, usual care control. Eligible study participants will be *N* = 450 parent-child pairs (youth ages 9–12 years and their parents/caregivers) recruited from participating BGCs. The 6-week in-person H_2_GO! intervention consists of 12 group-based sessions delivered by BGC staff and youth-led activities. An innovative feature of the intervention is the development of youth-produced narratives as a strategy to facilitate youth empowerment and parental engagement. Child outcomes include measured body mass index z scores (zBMI), beverage intake, and youth empowerment. Parent outcomes include beverage intake and availability of SSBs at home. Outcomes will be measured at baseline and at 2, 6, and 12 months. With a 75% retention rate, the study is powered to detect a minimum group difference of 0.1 zBMI units over 12 months.

**Discussion:**

Empowering youth may be a promising intervention approach to prevent childhood obesity through reducing SSB consumption. This intervention was designed to be delivered through BGCs and is hypothesized to be efficacious, relevant, and acceptable for the target population of low-income and ethnically diverse youth.

**Trial registration:**

ClinicalTrials.gov NCT04265794. Registered 11 February 2020.

**Supplementary Information:**

The online version contains supplementary material available at 10.1186/s12889-021-11660-5.

## Background

Over one third of U.S. school-age children are overweight or obese [1, 2] and at risk for diabetes, heart disease, and shorter life expectancies [3]. Reducing sugar-sweetened beverage (SSB) intake is a high-impact dietary target for obesity prevention, particularly among low-income youth and youth of color who have persistently higher SSB intake and obesity risk [4–6]. Data from the 2013–2014 National Health and Nutrition Examination Survey (NHANES) indicate that 63.5% of youth ages 6–11 years consumed ≥1 SSBs on a given day [7], with considerably higher SSB intake among low-income youth and Hispanic and non-Hispanic Black youth [4, 5, 7–9]. Low-income youth are also more likely to be heavy SSB consumers (≥500 kcals/day) than higher-income youth [9, 10]. Reducing SSB intake can lead to 8–11% reduction in energy intake among youth [4, 11–15]. Efficacious strategies to reduce SSB intake are therefore needed, particularly among low-income youth and youth of color who are high SSB consumers.

Empowerment approaches hold potential for catalzying positive behavior change in childhood obesity intervention contexts by building youth’s capacity to affect change in their lives and in the broader community [16, 17]. Empowerment-based health interventions seek to improve health behaviors and outcomes, particularly among low-income populations and communities of color, by helping individuals develop an ecological understanding of health and identify strategies for change that are relevant in the context of their lived experiences [18, 19]. There is growing body of evidence that indicates empowerment may mediate behavior change and obesity-related outcomes among youth [16, 17, 20, 21]. Prior youth empowerment interventions have demonstrated small improvements in diet, physical activity, and BMI among youth [22–24], though few have utilized rigorous study designs or examined empowerment as a mediator of intervention effects.

Cultivating youth narratives or stories is one empowerment strategy that may facilitate empowerment and health behavior change among youth of color [25–28]. Unlike traditional didactic approaches, the development of narratives within an empowerment framework engages youth in a transformative process by recognizing knowledge embedded within their personal stories and encouraging youth to take action [19, 29, 30]. The creation and sharing of narratives thus facilitates empowerment through elaborate message processing and personalization [27]. The use of narratives within other health interventions has shown success in behavior change, including improved diet, among adults [31–35]. Results from our pilot site-randomized study of *N* = 100 parent-child pairs indicated that our narrative-based youth empowerment SSB intervention (H_2_GO!) was associated with 6-month reductions in child zBMI and 6-month reductions in SSB intake and increases in water intake among children and parents [36].

The goals of this study are to test the efficacy of H_2_GO! among an ethnically diverse sample of youth through a cluster randomized controlled trial (parallel group) and measure empowerment as a mediator. We partnered with Massachusetts Allianc of Boys and Girls Clubs of America (BGCs) to develop and pilot test H_2_GO! [37]. BGCs are an ideal partner for this proposal due to their commitment to empowering youth to lead healthy lives and their potential to reach large segments of our target population (4 million school-age youth; 56% low-income) nationally. We hypothesize that child participants in the intervention sites will have reduced zBMI and reduced SSB intake compared to child participants in the comparison sites and that the intervention effect(s) will be mediated by youth empowerment. This paper describes the design and methods for the cluster randomized controlled trial of the H_2_GO! intervention.

## Methods/design

### Theoretical foundation

**Fig. 1:**
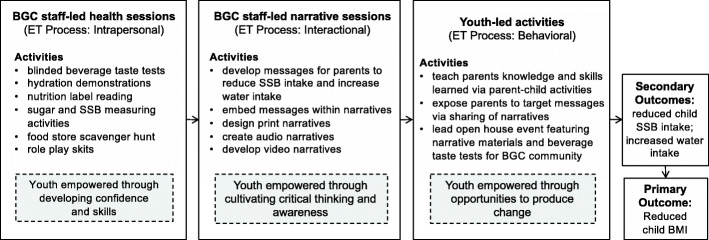
Intervention Conceptual Model by Empowerment Theory (ET) Processes

Our study applies Empowerment Theory (ET) [38] through intervention conceptualization, implementation, and measurement. Youth become empowered (e.g., gain mastery over their lives) as they develop critical consciousness, which occurs as they begin to fully understand the factors that shape their environment and in turn their behavior [39]. ET posits that youth empowerment occurs through three interrelated components or processes: 1) *intrapersonal* (development of youth beliefs, such as confidence and sense of agency to make a difference); 2) *interactional* (cultivation of critical awareness and critical thinking skills to help youth become independent decision makers and create situations aligned with their goals); and 3) *behavioral* (provision of opportunities for youth to practice skills in real life contexts and to take action to produce desired changes in their communities) [17]. Our intervention was developed for early and pre-adolescent youth ages 9–12 years, as children under 9 may not have the concrete operational development [40] needed for skills targeted in an empowerment intervention. The H_2_GO! intervention integrates the three ET processes to target SSB intake among youth through BGC staff-led sessions and youth-led activities (Fig. 1).

### Study setting and population

BGC is a national organization that provides affordable after-school programs for over 4 million school-aged youth (29% White, 27% Black, 24% Latino) across 4300 sites nationwide [41]. BGCs are an ideal community partner for youth empowerment interventions due to their commitment to youth empowerment and health promotion [42–48]. BGC sites will be recruited from the 40 BGC sites in Massachusetts, USA and selected for comparability in enrollment size and ethnic composition.

Parent-child pairs will be screened, recruited, and consented by study staff from participating BGC sites via in-person, phone, or web-based modalities. Study staff will obtain parental permission and verbal assent through three main ways: 1) verbal parental permission and verbal child assent in-person in the BGC setting; 2) verbal parental permission and verbal child assent over the phone using a REDCap form (completed and documented by study staff); and 3) online form using REDCap documenting parental permission and verbal child assent. *Child inclusion criteria are:* ages 9–12 years; current member at the BGC study site; able to understand and communicate in English; able and willing to provide consent; parental/caregiver permission to participate; has access to a wifi-enabled device at home (to allow for study participants to continue with intervention participation in the event the intervention needs to transition to online delivery due to COVID-19); and no medical condition that limits ability to change beverage consumption behaviors. *Parental/caregiver inclusion criteria are:* parent/caregiver to a BGC child member; 18 years or older; able to understand and communicate in English; able and willing to provide consent; and no medical condition that limits ability to change beverage consumption behaviors.

### Study design

This study is an unblinded parallel-group cluster randomized controlled trial with *N* = 10 BGC sites randomized to the intervention or comparison status. Geographic location is used as a stratification variable; sites within each stratum are spaced > 5 miles apart to minimize contamination bias. One site within each stratum will be randomly assigned to the intervention and the other to a wait-list, usual care condition. The randomization will be conducted by the project statistician using computer-generated random numbers. We opted to use a stratified sampling scheme to increase balance of BGC characteristics between study arms and include a representative sample of BGCs to increase generalizability.

**Fig. 2:**
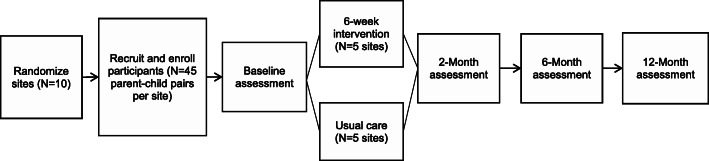
Study Flow Chart

After sites are randomized, participants will undergo recruitment and enrollment and complete in-person study assessments at baseline, 2, 6, and 12 months follow-up (see Fig. 2). Comparison group participants will have study assessments scheduled to match the timing of each intervention cycle. To enhance adherence to intervention protocols, the study team will complete intervention fidelity and process measures of intervention sessions using intervention attendance, recruitment, and retention rates, as well as an intervention fidelity checklist to score completion of intervention activities (0 = did not do this activity; 1 = partially completed; 2 = completed) across each of the 12 intervention sessions. The study team will also monitor intervention sessions and provide corrective feedback and ongoing trainings and refresher sessions as needed. Intervention fidelity monitoring and corrective feedback processes will be completed remotely via Zoom when study staff were unable to be present onsite BGC due to COVID-19 restrictions.

Study protocol and procedures, including adapatations and alternative plans that accommodated for COVID-19 restrictions and guidance for in-person and indoor activities, were approved by the Boston University Medical Center Institutional Review Board (protocol #H-39841). Any important protocol modifications will be communicated and approved by the investigators, the IRB, trial participants, the trial registry, and the study sponsor.

### Intervention development

The H_2_GO! intervention was designed to reduce SSB intake (recommended guideline of 0 SSBs/day) and promote replacing SSBs with water (recommended guideline of 5–8 cups/day) among 9–12 year old youth and their parents. Intervention materials, strategies, format, and content were previously pre-tested and refined based on youth, parent, and staff feedback and study staff observations on intervention feasibility, acceptability, and engagement in our pilot study [36, 37]. To prepare for the event that part or all of the intervention may need to be delivered remotely, the study team developed virtual curriculum content and activities (designed to be delivered via Zoom) to parallel the in-person activities. Study staff will train BGC staff to deliver the intervention through a remote-based initial training followed by quarterly booster sessions; this training will review curriculum and protocols for each intervention session through an intervention manual, provide practice of protocol implementation, and include delivery of feedback during mock sessions.

### Intervention condition

The 6-week intervention consists of 12 group-based weekly in-person sessions (1-h sessions twice a week) delivered by trained BGC program staff to BGC child study participants in the BGC setting during regular BGC hours (after-school on weekdays) and a culminating BGC community open house event for all youth and parent members. Intervention activities consist of three main components mapped onto the three ET processes:

1. **BGC staff-led health sessions for youth (intrapersonal)**: Youth will participate in weekly group-based interactive health sessions delivered by trained BGC staff. The primary objectives are to decrease SSB intake among youth and to promote water intake. Strategies to lead knowledge, confidence, and skill building activities targeting children’s SSB consumption have been previously pilot-tested in our study population (see Table 1 for session topics).
2. **BGC staff-led narrative sessions for youth (interactional)**: Youth will be guided by BGC staff to produce narratives featuring behavioral messages on beverage intake, applying knowledge and skills acquired from the health sessions. Narrative materials will build upon youth’s lived experiences, consist of youth’s own stories, and be developed by youth through a variety of mediums (e.g., print, audio, video). Staff will use pictorial-based analysis methods (e.g., SHOWed) to guide youth to critically reflect on narratives created [49].
3. **Youth-led activities empowering youth as change agents (behavioral)**: Youth will engage parents in critical dialogues by teaching parents knowledge and skills learned through weekly take-home activities and sharing of narrative materials produced, culminating in a youth-led BGC community event that will include a display of narrative materials through an art gallery format; live youth performances, a viewing of finalized narratives, and youth-led taste tests of non-sweetened beverages.

**Table 1:** Sample H_2_GO! Pilot Intervention Session Topics and Activities

Weekly BGC-staff led health sessions *Intrapersonal* (building confidence and skills)	Weekly BGC-staff led narrative sessions *Interactional* (critical thinking)	Weekly youth-led activities *Behavioral* (organized youth action)
1. *Water is Good for You!* (hydration demonstration)	Develop print narratives to promote water intake	Teach parents information and skills learned through parallel weekly parent-child activities Engage parents in critical dialogues on target behavioral messages through weekly sharing of narratives
2. *Re-Think Your Drink* (blinded taste tests of flavored water)	Develop print narratives to encourage non-SSB alternatives	
3. *Find the Facts* (label reading, SSB measuring activity)	Develop print narratives to explain how to identify SSBs	
4. *Explore the Corner Store* (scavenger hunt of SSBs and non-SSBs)	Develop audio narratives to explain how to identify SSBs	
5. *Water, Water, Everywhere* (role play skits to find ways to drink water)	Develop video narratives to find opportunities to drink water	
6. *SSB Triggers* (role play skits to manage SSB triggers	Develop video narratives to manage SSB triggers	
**Culminating youth-led BGC community event featuring display of narratives and flavored water taste tests** *Behavioral* (organized youth action)		

Child participants will also receive a reusable water bottle and a pictorial intervention booklet developed by the research team, which includes intervention activity worksheets, parent-child take-home activities, and beverage consumption tracking and goal-setting sheets. Activity worksheets will be completed by participants during intervention sessions, and parent-child take-home activities will be completed following each session. Sample session topics and activities are summarized in Table 1. Additional details on intervention strategies and intervention session activities have been previously described [36, 37].

### Comparison condition

Parent-child pairs in comparison sites will receive usual care (standard BGC programming) during the study and the intervention upon study completion. BGCs deliver health and life skills programs each year on a variety of topics determined by each site (e.g., cyber-safety, substance use prevention). Comparison sites will continue to implement such programs but will refrain from implementing obesity-specific programs. Upon completion of data collection, the study team will train BGC staff in comparison sites to deliver the H_2_GO! intervention and provide intervention toolkits and protocols.

### Measures

#### Primary outcome

The primary outcome is change in child zBMI (number of standard deviations by which a child differs from the mean BMI of children of the same age and sex) over 12 months, an appropriate measure of that allows for comparing children of different ages over time as they grow [50]. Children’s height and weight will be used to calculate BMI, age- and sex-specific BMI percentiles, zBMI, and standard BMI-based weight status categories using CDC growth charts [51]. Trained BGC and study staff will take the average of two measurements of children’s height and weight using a stadiometer and a medical quality scale, respectively. Children will be measured in a private setting wearing light clothing (e.g., without shoes and heavy outer layers). Secondary outcomes and covariates for parent and child participants are summarized in Table 2 and are based upon validated instruments and surveys [52–61].

**Table 2:** Secondary Outcome and Covariate Measures

Child Outcomes (self-report)	
SSB and water consumption *(secondary outcomes)*	A 15-item beverage intake questionnaire (BEVQ) will be used to assess frequency and amount of SSB and water consumption and energy intake (kcalories) from SSBs. The BEVQ has demonstrated reliability and validity in assessing beverage intake against 24-h recalls among youth [52].
Beverage intake self-efficacy	Self-efficacy to reduce SSB intake and increase water intake will be assessed using items from a nutrition self-efficacy scale validated among youth ages 8–11 years [53].
Diet	Frequency of consuming specific foods/food groups (e.g., vegetables, fruits, fast food, desserts) will be assessed using items from the School Physical Activity and Nutrition (SPAN) monitoring system [54]. These items have been validated for use by children in grades 4 and higher and were selected for their brevity and low response burden.
Physical activity	Time spent outdoors [55] and number of days during the past week children participated in ≥ 60 min of moderate-to-vigorous physical activity [56] will be used to assess child physical activity.
Screen time	Hours/day watching TV away from school (on TV or through a mobile or computer device) and hours/day playing video or computer games or using a computer for something other than schoolwork (including time on the Internet, instant messaging) away from school [54] will be used to assess screen time.
Youth empowerment	The 8-item Sociopolitical Control Scale for Youth (SPCS-Y) [57], which has been validated as a measure of empowerment among urban youth [58], will be used to assess youth empowerment. The SPCS-Y uses a 10-point, phrase completion response format and consists of a leadership/competence subscale and a policy/social control subscale [59].
Socio-demographics	Child gender, age, and race/ethnicity will be assessed via brief survey items.
**Parent Outcomes (self-report)**	
SSB and water consumption	Items from the beverage intake questionnaire (BEVQ-15) [60] assessing frequency and amount of beverages consumed will be used to assess SSB and water intake and SSB energy intake. The BEVQ-15 has been validated against 24-h dietary recalls among adults, including low-literacy populations [60].
Home SSB availability	One survey item assessing how often SSBs are available at home (5-point Likert scale ranging from “never” to “always”) [61] will be used to measure SSB home availability.
Socio-demographics	Gender, age, race/ethnicity, education, income, occupation, and child eligibility for free/reduced priced lunch will be assessed via survey items.

### Statistical approach and power

Our sample size calculations (*N* = 45 parent-child pairs per each of the 10 sites) are based on the primary hypothesis that child participants in intervention sites will have reduced zBMI compared to child participants in comparison sites over 12 months. Our pilot study showed an effect size of 0.22 zBMI units over 6 months [36]. We powered our analysis to detect a minimum effect size of 0.1 units over 12 months (a conservative approach considering possible attenuation of effects over the doubling of study time period) and anticipate a 0.15 SD in both groups. A 0.1 effect size is reasonable for populations inclusive of healthy weight children; of studies targeting school-aged children including those of healthy weight, randomized SSB interventions have yielded 0.0–0.13 unit differences in zBMI over 12–18 months [62, 63], and childhood obesity prevention interventions have yielded 0.10–0.16 unit differences in zBMI over 12 months [64, 65]. We assume an intra-class correlation coefficient of 0.05 [66–68]. With α = 0.05 and a 75% retention rate, we can detect ≥0.1 unit difference in zBMI with over 80% power and 0.2 unit difference with over 90% power by enrolling a total of 450 parent-child pair participants.

### Recruitment and retention

We will utilize the following participant recruitment approaches previously tested in our pilot study (identify eligible child participants through the BGC electronic enrollment record system and in collaboration with BGC staff), which yielded > 95% recruitment rate in our pilot study. Proposed strategies to retain participants include collecting multiple forms of contact information from parent/caregiver participants at baseline, scheduling pre-determined study assessments at dates/times convenient for participants, providing options for online or phone follow-up assessments, implementing a tracking and reminder system, offering assistance and support for participating in study assessments, using email and social media contact approaches, and providing incentives of $20 gift cards per assessment.

### Data management and confidentiality

A number of procedures will be utilized to maximize data integrity and quality control procedures for data collection and data entry. Quality control measures implemented by the project data analysists will include detailed and unabmigious specifications for completion of each of the data collection forms, including rules for coding skipped questions, missing data, and/or refusals. Interim incremental data reviews will be performed on all data to compare data collectors and determine variations among observers in responses to questions on data forms. Computer algorithms will be written to check logic and identify internal inconsistencies.

Password-protected study databases and locked offices will be used to maintain data security to the highest extent possible. The key to identification of subjects will be kept in a separate and secure location. These data will be stored and managed on a secure server in a HIPAA-compliant data center with daily back-up. Data entry will be through the REDCapTM Data Capture System (Vanderbilt University). All databases and analytic files will be contained within the Biostatistics and Epidemiology Data Analytics Center (BEDAC) secure environment with access controlled through user-specific login and passwords.

### Planned statistical analysis

Distributions, descriptive statistics, and missing values will be examined for child zBMI. Bivariate analyses will compare intervention and comparison group characteristics using chi-square tests for categorical variables and t-tests for continuous variables. Any statistically notable (*p* < 0.1) imbalances that arise between groups will be adjusted for in multivariable analyses. Analyses will utilize an intent-to-treat approach, with each participant enrolled in the intervention site analyzed as part of the group.

To test our primary hypothesis that children in intervention sites will have decreased zBMI compared to children in comparison sites, we will apply generalized linear mixed models. The hierarchical structure of the data (repeated measures on youth nested within sites) will be modeled by including participant- and site-level random intercepts. A more complex covariance structure for repeated measures over time on participants (e.g. unstructured covariance) will be incorporated into the analysis if necessary. The main predictors of the model include study condition (intervention vs. control), all time points, and the interaction term between study condition and time points. Intervention effects will be evaluated over the entire study period by testing the interaction term of time and study condition, and by specifically comparing change from baseline to 12 months via a statistical contrast. We will also compare changes from baseline to earlier time points using this approach. Covariates associated with child zBMI (e.g., gender, race, other dietary intake, physical activity) will be examined for balance between groups and incorporated into multivariable analyses if imbalances (*p* < 0.1) occur. Potential moderating effects of gender, age, and race/ethnicity on intervention effects will be examined via a three-way interaction with the moderator, the study condition indicator and time. Should significant interaction terms emerge from the data, we will analyze and report intervention effects by appropriate subgroups (e.g., males vs. females). Participants with incomplete follow-up can be included in analyses, which are valid under a missing at random assumption. We will assess the likelihood of nonignorable missing data using a pattern mixture model.

To test our secondary hypothesis that children in intervention sites will have decreased SSB intake and increased water intake than children in control sites over 12 months, we propose to compare SSB and water intake using linear mixed models and similar approaches described in the primary hypothesis testing. Comparisons of group mean changes from baseline to 2 and 6 months will also utilize this approach. We will additionally examine changes in child self-efficacy, parental beverage intake, SSB home availability, and spontaneous changes in other BMI-related behaviors (other dietary intake, physical activity, screen time, sleep) over time and by study condition using methods described above. Finally, for our mediation hypothesis, we will first examine whether the pattern of change in youth empowerment over time differs by study condition via a statistical interaction using a linear mixed model. Next, we will examine whether patterns of change over time in primary and secondary outcomes depend on level of empowerment by incorporating an empowerment by time interaction. Finally, we will incorporate both interaction terms (study condition by time and empowerment by time) to determine if the intervention effect is partially or fully mediated by empowerment.

### Data safety monitoring board

Given the minimal risk involved, a full Data and Safety Monitoring Board was not deemed necessary. The PI and project team will be involved in ongoing monitoring of the trial and will meet annually to review study progress, data quality and safety.

### Dissemination plan

We plan to engage past study participants and community members in a series of community presentations regarding study results. We will post results in our project website, including study description, briefs with findings, and publications, as well as share findings to BGCs via newsletters, social media outreach, and local, regional, and/or national presentations. If the intervention is shown to be efficacious, we plan to create a dissemination package for our community partners that includes: manuals describing intervention components; procedures and resources for implementation and evaluation; staff training modules; and information regarding intervention feasibility and acceptability to inform adoption. Within the broader scientific community, we plan to submit study findings for presentation at national conferences and to publish study results in relevant peer-reviewed journals.

## Discussion

Reducing SSB intake is a critical target (and one of many needed) for childhood obesity prevention. The H_2_GO! intervention targets SSB intake through youth-produced narratives as a strategy to facilitate youth empowerment and parental engagement. The result is a novel SSB intervention that strategically engages youth in developing confidence, skills, and opportunities to affect their broader circumstances through cultivating and sharing their stories in the family and community settings. Our study also aims to measure youth empowerment as a mediator in the context of a childhood obesity prevention trial. Establishing this association will build further evidence of utilizing youth empowerment strategies to improve obesity-related behaviors and outcomes among youth.

We partnered with BGCs, a system of affordable afterschool care that reaches our target population (56% low-income; 33% White, 30% Black, 23% Hispanic) nationally [42]. BGCs are an ideal setting and leverage point for childhood obesity prevention programs, given that SSB intake and childhood obesity are major problems that have yet to be sufficiently addressed in this population and setting. The H_2_GO! intervention was designed in collaboration with BGCs for implementation through BGCs, a setting that has national reach and infrastructure to support our intervention model. The resulting H_2_GO! intervention aligns with the BGC goal of empowering youth to lead healthy lifestyles and has high potential for sustainability and dissemination.

## Supplementary Information

**Additional file 1.**

**Additional file 2.**

## Data Availability

Not applicable.

## Abbreviations

BMI: Body mass index
BGC: Boys and Girls Clubs of America
ET: EmpowermentTheory
SSB: Sugar-sweetened beverages
zBMI: Body mass index z score
