# Supplementary material for: A youth empowerment intervention to prevent childhood obesity: design and methods for a cluster randomized trial of the H2GO! program
Source: BMC Public Health. 2021 Sep 15;21:1675. doi: 10.1186/s12889-021-11660-5 (PMC8441230; doi:10.1186/s12889-021-11660-5)
Supplement: Supplementary file 1 — Additional file 1. [file 12889_2021_11660_MOESM1_ESM.pdf]

Empowering youth and families to develop healthy habits

Boston University School of Public Health

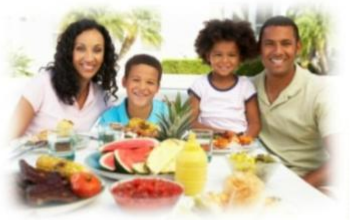

Researchers at Boston University School of Public Health want to learn more about helping families with healthy habits.

This research study is for children ages 9-12 years and parents. Research is always voluntary!

What would happen if I took part in the study?

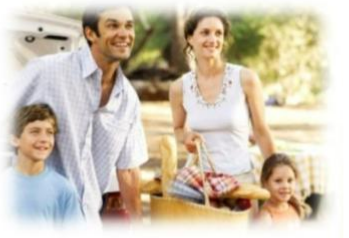

Children:
If you decide to take part in the research study, you would:

- attend a 6-week health program at the Boys & Girls Club
- fill out a survey before the study, 2 months later, 6 months later, and 12 months later
- have your height and weight measured before the study, 2 months later, 6 months later, and 12 months later

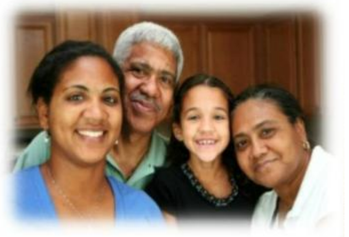

Parents/Caregivers:
If you decide to have your child take part in the research study and decide to take part in the research study as a parent participant, you would:

- fill out a survey before the study, 2 months later, 6 months later, and 12 months later

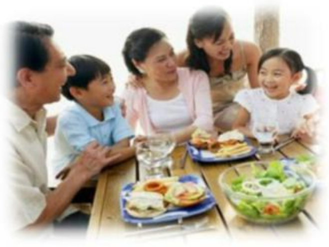

Children and parents who take part **will each receive $20 Amazon gift cards** each time they complete a study assessment to thank them for their time.

There may be possible benefits for you if you take part in the study, such as learning more about healthy habits and improved eating habits.

To take part in this research study or for more information, please contact Selenne Alatorre at (617)-358-1571 or at salatorr@bu.edu

The principal researcher for this study is Dr. Monica L. Wang at Boston University School of Public Health.

BMC and BU Medical Campus IRB
IRB NUMBER: H-39841
IRB APPROVAL DATE: 10/08/2020
